# Contralateral extradural hematoma following decompressive craniectomy for acute subdural hematoma (the value of intracranial pressure monitoring): a case report

**DOI:** 10.1186/1752-1947-8-153

**Published:** 2014-05-16

**Authors:** Lucas Crociati Meguins, Gustavo Botelho Sampaio, Eduardo Cintra Abib, Rodrigo Antônio Rocha da Cruz Adry, Richam Faissal El Hossain Ellakkis, Filipe Webb Josephson Ribeiro, Ângelo Luiz Maset, Dionei Freitas de Morais

**Affiliations:** 1 Faculdade de Medicina de São José do Rio Preto (FAMERP), Residente do Serviço de Neurocirurgia, Hospital de Base, São Paulo, Brazil; 2 Faculdade de Medicina de São José do Rio Preto (FAMERP), Coordenador do Setor de Emergência do Serviço de Neurocirurgia, Hospital de Base, São Paulo, Brazil; 3 Faculdade de Medicina de São José do Rio Preto (FAMERP), Chefe do Serviço de Neurocirurgia, Hospital de Base, São Paulo, Brazil; 4 Rua Pedro Palotta, 101/31B. Jardim Maracanã, 15092205 São José do Rio Preto, São Paulo, Brazil

**Keywords:** Acute subdural hematoma, Decompressive surgery, Extradural hematoma

## Abstract

**Introduction:**

Decompressive surgery for acute subdural hematoma leading to contralateral extradural hematoma is an uncommon event with only few cases previously reported in the English medical literature.

**Case presentation:**

The present study describes the case of a 39-year-old White Brazilian man who had a motorcycle accident; he underwent decompressive craniectomy for the treatment of acute subdural hematoma and evolved contralateral extradural hematoma following surgery.

**Conclusion:**

The present case highlights the importance of close monitoring of the intracranial pressure of severe traumatic brain injury, even after decompressive procedures, because of the possible development of contralateral extradural hematoma.

## Introduction

Traumatic brain injury (TBI) represents an important life-threatening disease. In 2009, the US Centers for Disease Control and Prevention estimated that at least 2.4 million emergency department visits, hospitalizations, or deaths were related to a TBI, either alone or in combination with other injuries [1]. Posttraumatic intracranial mass lesions are commonly seen after severe TBI and are usually involved in the pathophysiology of intracranial hypertension. They may vary from extra-axial mass lesions (acute subdural hematomas, [ASDHs], and extradural hematomas, [EDHs]) to intraparenchymal mass lesions (contusions and intracerebral hematomas) [2-5]. However, EDH following decompressive surgery for ASDH is an uncommon situation with only few cases previously reported in the English medical literature [6-10].

The present report describes the case of a Brazilian man who evolved EDH following decompressive surgery for ASDH. We review similar cases previously published.

## Case presentation

**Figure 1:**
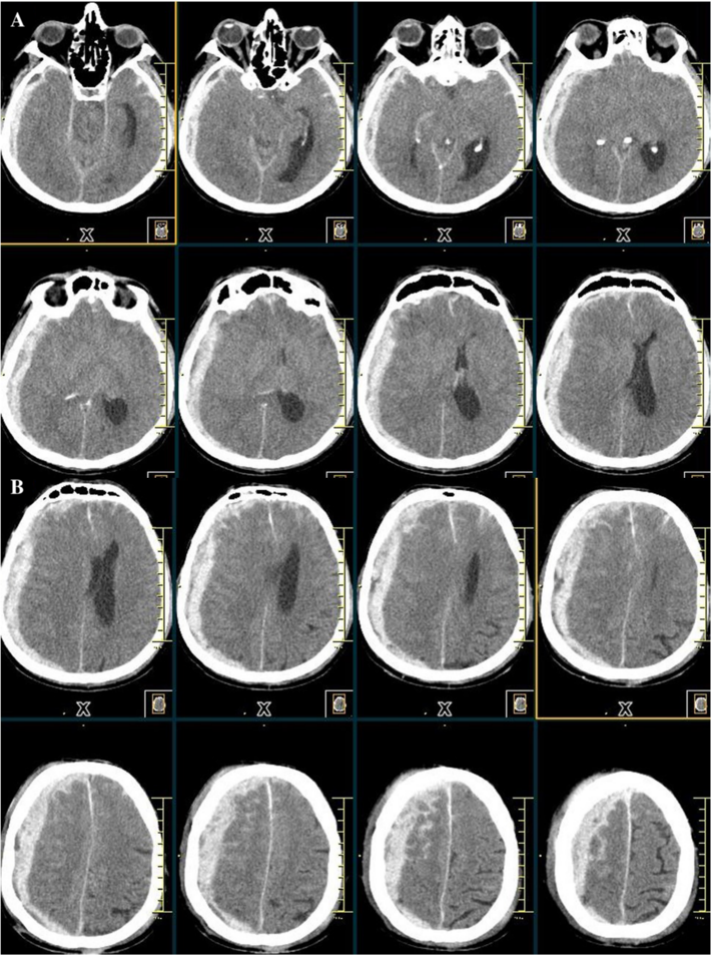
(A/B): Admission computed tomography showing right acute subdural hematoma.

**Figure 2:**
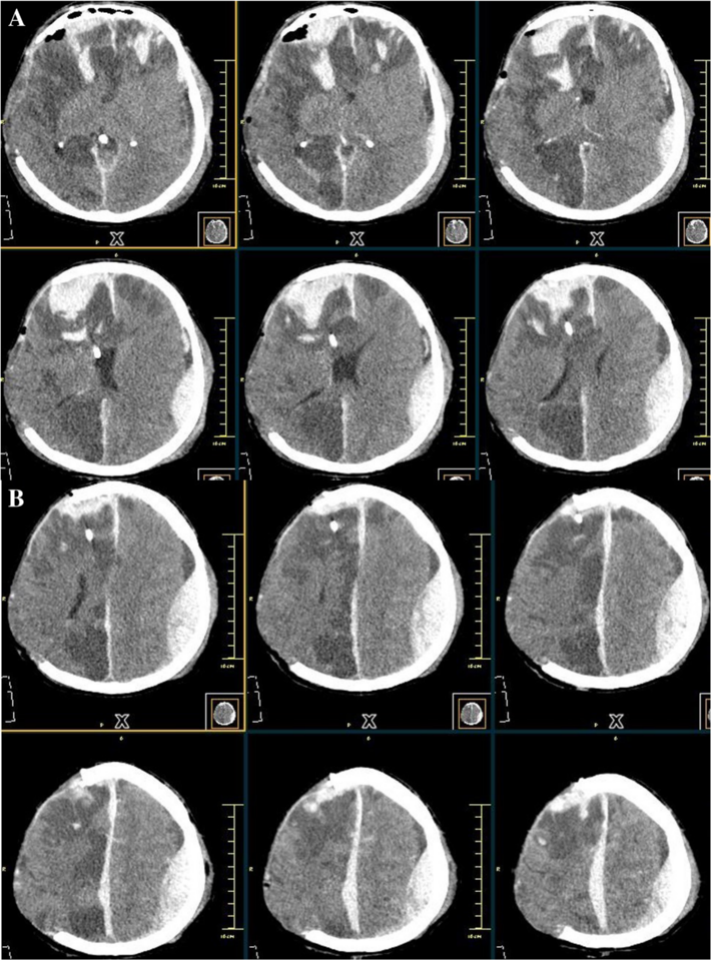
(A/B): Computed tomography following decompressive surgery showing contralateral extradural hematoma.

**Figure 3:**
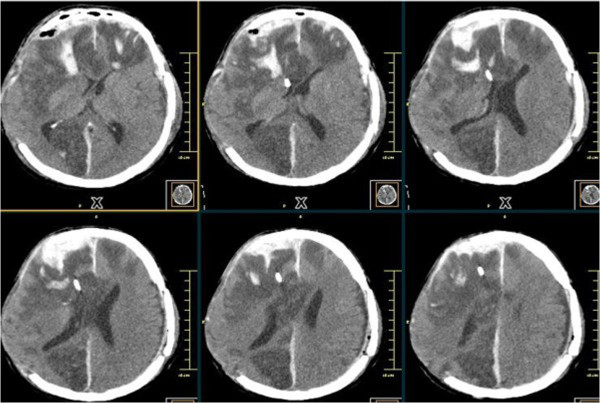
Computed tomography following drainage of extradural hematoma.

A 39-year-old White Brazilian man was admitted to our Emergency Department after a motorcycle accident on a highway. Although he was hemodynamically stable, a neurological assessment revealed a Glasgow Coma Scale (GCS) of six points and right eye mydriasis. No clotting dysfunction was detected by laboratory test. Computed tomography (CT) of his brain revealed right side ASDH, hemispheric edema and midline shift of 15.7mm with compression of his right lateral ventricle (Figure 1). He was immediately transferred to the Operating Room and a right decompressive craniectomy was performed. He was kept under sedation and his intracranial pressure (ICP) was continuously monitored in the intensive care unit. On the first postoperative day, he started to present elevated ICP refractory to hyperventilation and osmotic therapy. A new CT was then obtained and showed a large contralateral EDH (Figure 2) and frontal hemorrhage associated with catheter insertion. He was taken again to the Operating Room and surgical evacuation of the hematoma was performed. A brain CT following the second operation, revealed no residual EDH (Figure 3). He evolved hemodynamically unstable within the first 48 hours and no neurological improvement was observed after weaning sedation. He died on the ninth postoperative day.

## Discussion

Posttraumatic intracranial mass lesions are commonly seen after severe TBI and are usually involved in the pathophysiology of intracranial hypertension. ASDH is frequently associated with other intracranial abnormalities, and only 30% to 40% of all ASDHs requiring surgery are isolated lesions [11,12]. The most frequently associated intracranial lesions are contusions and intracerebral hematomas, associated EDHs are noted in 6% to 14% of patients [12,13]. However, EDH following decompressive surgery for ASDH is an extremely uncommon situation with only 38 cases previously reported in the English medical literature [6-10].

Several neurosurgical procedures have been reported to be associated with contralateral EDH, such as ventriculoperitoneal shunt insertion and evacuation of acute and chronic ASDH [6-10,14,15]. Shen *et al.*[6], in 2013, estimated that the percentage of occurrence of EDH contralateral to the site of drainage of ASDH was 2.4% according to the published data. Most of the patients were male, with an average age of 35 years and with a main mechanism of traffic accident [6]. The present report describes the case of an adult who had a motorcycle accident who was admitted to our Emergency Room showing signs of severe neurological damage, with GCS of six points and anisocoria.

Many signs have been proposed as alert hints to the detection of contralateral EDH following drainage of ipsilateral ASDH, such as intra-operative brain swelling, postoperative neurological deterioration, pupillary dilation contralateral to the site of ASDH, grand mal seizure and intractable elevated ICP [8,14,15]. In our case, the patient was admitted in poor neurological status and was continuously sedated. ICP monitoring revealed increased ICP from the first postoperative day and was refractory to all initial clinical maneuvers. A postoperative CT showed the presence of a large contralateral EDH. As already highlighted by other authors, on the presentation of “red flags” immediate CT is recommended because it may lead to urgent evacuation surgery of these life-threatening mass lesions [7,16].

The pathophysiology involved in the formation of delayed contralateral EDH following decompressive surgery is not fully understood, but may include loss of tamponade effect, vasomotor mechanisms, and coagulopathy, with the main cause appearing to be the upsetting of the equilibrium of the damaged vessels and the reactive ICP [17]. In the present case, although a linear fracture ipsilateral to the ASDH had been identified on the admission CT, no other structural abnormality was found during drainage of contralateral EDH, making us believe that the main mechanism involved in our case was the loss of tamponade effect following decompressive surgery and microvascular ruptures in the virtual epidural space.

## Conclusions

In conclusion, the present case highlights the importance of close monitoring of the ICP of patients who have severe TBI, even after decompressive procedures, because of the possible development of contralateral EDH. Although most instances of increased ICP after ASDH drainage seem to be associated with brain swelling, these phenomena should raise the suspicion of evolution of contralateral hematoma.

## Consent

Written informed consent was obtained from the patient’s next of kin for publication of this case report and any accompanying images. A copy of the written consent is available for review by the Editor-in-Chief of this journal.

## Competing interests

The authors declare that they have no competing interests.

## Authors’ contributions

LCM, GBS, ECA, RE and FR participated on the surgeries of the patient. All authors helped to draft the manuscript. All authors read and approved the final manuscript.
